# Optimization of two-wheeler Bike engine among three different designs using design of experiments

**DOI:** 10.1016/j.heliyon.2024.e34081

**Published:** 2024-07-05

**Authors:** Manish Dadhich, Vikas Sharma, Gaurav Jain, K. Loganathan, V. Karunakaran, Mohamed Abbas, P. Subhashini

**Affiliations:** a Maxbrain Technologies Private Limited, Jaipur, Rajasthan, India; b Department of Electrical Engineering, Poornima College of Engineering, Jaipur, Rajasthan, India; c Department of Mathematics and Statistics, Manipal University Jaipur, Jaipur-303007, Rajasthan, India; d Centre for Artificial Intelligence and Machine Learning, Department of CSE-AI and ML, Sri Eshwar College of Engineering, Coimbatore, India; e Electrical Engineering Department, College of Engineering, King Khalid University, Abha 61421, Saudi Arabia; f Department of Computer Science and Information Technology, MLR Institute of Technology, Hyderabad, Telangana, India

**Keywords:** Heat transfer coefficient, Nusselt number, DOE

## Abstract

This research presents the results of a fin heat transfer study of three different models of the motorcycle engine. The objective of this study is to minimize the external body temperature because the optimum design will be found when the heat transfer rate is maximized. The study obtains the procurement of a balance between the outer boundary temperature due to convective heat transfer and air-cooled fin design. Heat transfer coefficient varies according to wind velocity (Km/hr) and Nusselt Number. The analysis was performed on three different engine models, named A, B and C. The optimum design was design B through simulation which has lower temperature gain, lower deformation and lower normal stress. DOE (Design of Experiments) was performed on the optimum design of the engine among all with three parameters thickness of fin, size of fin, and shape of fin, and again analysis was performed according to DOE cases. The material used for manufacturing the models was aluminum alloy 6061 which has a thermal conductivity of 200 W/mK. The study was performed on the designed models by taking the outer boundary temperature of 750^o^C. The heat transfer coefficient was about 77.28 W/m^2^K at 40 Km/h velocity.

## 1 Introduction

Fins have significant uses in a variety of industries, including heat exchangers, heat pipes, motorcycle engines, refrigerators and chemical processing systems, the topic of heat transfer using fins has drawn the attention of numerous researchers. When heat transfer rates are optimized, power consumption is reduced and efficiency is raised for devices like computer chips and car engines, among other things. Usually, the purpose of heat transfer enhancement devices is to speed up the rate of heat transfer from the heat source to the surrounding air. To improve heat transfer from the primary surface to the convective-radiative environment, fins are used. An ideal shape design became more crucial since, in the majority of applications, weight and material costs are the primary design factors. Variation in fin outer temperature and deformation due to heat and mass transfer is based on temperature and humidity ratio difference [1]. Longitudinal fin optimization with volumetric heat generation by conduction along the fin and dissipated from convective heat loss on the surface of the body via natural convection to the ambient environmental conditions [2]. Increase the heat transfer coefficient range with perforated fins of 12 mm perforation diameter at the angle of orientation is 45° in this case of steady state heat transfer [3]. Nusselt number enhancement ratio and embossed fin effectiveness are the key parameters of heat transfer performance for the values of impression angle vary between 30° to 90° and impression pitch is caught on as 12 mm, 16 mm and 20 mm at different heat input rates [4,5]. Producing the strong vortex flow increases dimples near the wall turbulent mixing level to raise the convective heat transfer in the channel [6]. Fin array performance is based on the variation in pitch, thickness and fin material [7]. In the air-cooled engine cooling mechanism is dependent on the cylinder body, cylinder block and insufficient heat removal from the surface of the cylinder block produces more thermal stress, and lower thermal efficiency [8]. Pekkaya et al. [9] utilized artificial neural networks (ANN) in order to find out and model the crucial factors that affected the sales volume of the steel and iron industry. The results were also compared using an ordinary least squares regression model, and a thorough conclusion was reached using grey relational analysis (GRA) based on the ANN results. The variables with the greatest degree of influence on the iron and steel company's sales were, according to the results, the USD/TL exchange rate, product prices, and interest rates, in descending order. According to the study, the ANN performed better in terms of prediction accuracy than traditional regression models. Furthermore, GRA was discovered to be an effective method for combining insights from various ANN models according to their individual performance levels.

Cuenca and Bermeo [10] carried out a finite element study on internal combustion engines at various loads in order to determine the structured dimensions to withstand these loads. For structural optimization, ANSYS-Workbench software was used. The results demonstrated that in order to obtain a suggested design life of 25 years, the minimum thickness for both the flange and the web of this structural element must be 12 mm. Another combination of thicknesses failed for the design life requirements. Fontanesi and Giacopini [11] analyzed and optimized the internal combustion engine using the Finite Element Method and Computational Fluid Dynamics techniques. CFD and experimental results of the engine were in good agreement. In the low-cycle fatigue region, an energy-based multi-axial criterion tailored for thermal fatigue is used, while well-established multi-axial stress/strain-based criteria are used to examine the high-cycle fatigue regions of the engine head. The suggested technique provides a precise thermo-mechanical characterization of engines under real life-cycle running circumstances and demonstrates highly good results in terms of point-wise identification of potential engine breakdowns.

Haiqiang et al. [12] conducted a failure analysis-based study on engine mounting bracket. A nonlinear finite element method and other analysis like chemical composition, failure morphology, micro-structural inspection etc. have been carried out in order to ascertain the reasons for the failure. According to the FEM findings, the maximum mises stress is somewhat greater and was located exactly where the crack first begins, at the bracket's fillet. The analysis demonstrated the optimized structure with a 20 % reduction in maximum stress. Makhutov et al. [13] carried out numerical, experimental and analytical studies in order to determine the strength, life and integrity of rocket space engines. For the analysis of the engine physical and mathematical modeling was carried out. Finite elements analysis was performed on the engine parts to calculate stress and strain. Finally, the safety factors for the engine parts were determined.

Jayanthi et al. [14] used the finite element analysis in order to optimize the compressor disc of an aero engine. Simulations were carried out in order to investigate the strain, load, temperature and flow behavior of the material. Based on the simulation results it was advised to upset a cylindrical billet in two stages utilizing a hydraulic press and die forging with a pneumatic hammer. In order to find out the natural frequency of the engine chassis Akmal and Bharathiraja [15] utilized the Finite Element Analysis. In this investigation, the modal analysis was carried out in which the natural frequencies were spotted in different modes in undamped and damped situations and the comparison of displacement was done. Results demonstrated that the elastomeric material on the engine behaved as a damper and reduced the deformation by 26 % and displacement by 17 %.

Sharma et al. [16] carried out an analysis of the piston engine coated with ceramic. The material of the piston was aluminum and silicon and was coated with Lanthanum Cerate (La_2_Ce_2_O_7_). The distribution of the temperature was investigated and was compared with an uncoated piston engine. A finite element analysis investigation utilizing ANSYS workbench software was performed. During the investigation, the coating thickness was varied from 0.4 to 1.6 mm to analyze the distribution of the temperature. Results demonstrated that at the top surface, the increment in the temperature was seen with the increase in coating thickness. Due to lower thermal conductivity, Lanthanum cerate showed better performance as a ceramic material in comparison to magnesium zirconate. Kirthana and Nizamuddin [17] did a topology optimization of a bracket mounted on an engine of Chevrolet beat. To carry out the finite element analysis tools like CATIA V5R20 and Hyper works were utilized. The main of the analysis was to reduce the weight of the bracket mounted on the engine by changing the design and the material. In order to determine the optimal model under specified parameters, stresses and weights were calculated and compared for various material layouts and designs.

Liu et al. [18] studied the thermo-physical properties of engine piston such as thermal conductivity, thermal diffusion coefficient, and specific heat made from 3 different Aluminum–Silicon alloys. In this study, the Cu and Ni's influence on the materials' thermo physical properties were investigated. Using the finite element analysis, the temperature distribution on the piston was studied under variable operating conditions. Experimentally the piston temperature was measured by the hardness plug method. Both the results of the experiments and the finite element method were in good agreement. The results demonstrated that as temperature rises, aluminum alloy piston material's thermal conductivity rises as well. Moreover, elevated thermal conductivity has the potential to lower the piston head's surface temperature. The inclusion of alloying metals like Cu and Ni raised the piston's surface temperature and decreased the thermal conductivity of aluminum alloy materials. Consequently, the heat resistance and high-temperature performance of piston may be controlled by appropriately adjusting the concentration of alloying elements. In order to anticipate the structural failure of exhaust manifolds, Yan et al. [19] used a coupled CFD-FEM technique. They discovered that high cycle fatigue collapse was caused by the first natural frequency. But in this instance, a normally aspirated engine was taken into account, and plastic stresses were not the main factor. In the investigation of the thermomechanical behavior of an exhaust manifold for a petrol engine with turbocharging, Chen et al. [20] developed an approach to model the component's structural failure as a result of plastic deformations.

Lorenzini et al. [21] carried out a static mechanical characterization of the materials involved at high temperatures with the goal of determining an appropriate alloy and its mechanical properties that would be helpful in feeding the Ferrari Engine's numerical models. The designed methodology takes into account the material's elastoplastic behavior under high-temperature cycles and suggests a damage criterion for thermal fatigue investigation. The methodology appears to be well correlated with the experimental evidence, thus limiting the number of tests necessary for the approval of the component. A novel yttrium barium zirconate (YBZ) coating was applied to a diesel engine piston, and the results of a thorough thermo-mechanical analysis were compared with those of other thermal barrier coatings (TBCs) with different thicknesses by Khan et al. [22]. The findings showed that when different TBCs were applied, the piston substrate surface temperature significantly decreased, with YBZ coating outperforming the others. The YBZ-coated piston's 0.2 mm coating showed notable reductions in temperature and thermal stress of 15 % and 10.3 %, respectively, improving the durability of the piston. The unique YBZ coating's superior performance over other TBC materials may be explained by its more elastic and stable thermal characteristics as well as its lower thermal conductivity.

Chen et al. [23] examined a natural gas engine's exhaust manifold failure from a material and structural standpoint. Material tests and data analysis were used to first perform the material analysis, which included mechanical and chemical properties. The exhaust manifold's temperature and thermal stress under heat shocks were then determined using the finite element method. Lastly, it was discussed how cracks start and spread as well as how to improve exhaust manifold design. The exhaust manifold's maximum temperature was recorded at 715 °C, and the maximum thermal stress measured was 455 MPa, according to the results. Thermal shocks caused the yield deformation. Additionally, the possibility of shrinkage pores was raised by the abrupt structural change at the critical induced thermal stress area. Consequently, the fatigue failure at low cycle was the cause of exhaust manifold failure. In order to improve the accuracy of the blade material of a turbojet aircraft engine Merculov et al. [24] utilized the finite element analysis. In the analysis, the analysis was performed for the striking of the bird with the engine. The simulation results were compared with the experimental results. In the analysis, the main focus was the centrifugal forces and loads that impacted the bird action on the blade under static conditions. Material behavior and strength properties utilizing the stress-strain characteristics were evaluated. The simulation results were in good agreement with the experimental results.

Liu et al. [25] performed a theoretical investigation in order to determine the thermo-mechanical condition of the piston of a diesel engine. For the analysis, the piston, empirical model and formulas were used to evaluate the stress fields, temperature and other boundary conditions. The finite element method was employed to compute the fatigue life along with stress loading. Additionally, the finite element method was used to simulate the harsh conditions and accelerated life testing was used to confirm the results. A relationship was established among the stress and piston life under thermal-mechanical coupling, mechanical and thermal load. The outcomes showed that various coupling conditions were in good agreement with the various mathematical models. Vardaan and Kumar [26] carried out a finite element analysis of the assembly consisting of cam and follower using ANSYS software. In this analysis, grey cast iron and structural steel materials were used. Contact pressure and stress were analyzed during the analysis. The outcomes demonstrated that contact pressure and stress increased linearly for both materials. The grey cast iron showed that the maximum values of contact stress and pressure were lower. Therefore, when it comes to building the cam and follower pair of IC engine, it was a better option than structural steel.

Zhu et al. [27] determined the fundamental reasons behind the wear failure of the oil hole in the third crankpin of an internal combustion engine with six straight-six cylinders made up of 42CrMo material. A thorough examination of this wear phenomenon was carried out using finite element analysis and experimental studies. The findings showed that the main cause of the abrasive particle wear in the third crankpin region is the entry of contaminated particles and grinding debris into the contact surface between the bearing shell and the third crankpin through the oil passage. The results of the finite element simulation showed that the most prominent deformation occurs at the center of the oil hole of the third crankpin. Utilizing Finite Element Analysis, Vidya et al. [28] investigated the thermo-mechanical analysis of composite cylinder liners made of boron-aluminum, carbon-carbon, silicon-carbide, graphite-phenolic, carbon-polyimide and carbon-phenolic to identify the stresses i.e. hoop, radial, and longitudinal. When the bottom and top of the cylinder linear area are constrained as boundary conditions, and both temperature and pressure of 1750K and 60 Nm^−2^ are applied in the same area, the carbon-carbon composite performed well.

In the diesel engine, Wahab et al. [29] examined the stress behaviors of cast iron and alloy steel with different thicknesses varying from 6.93 to 13.13 mm under the influence of thrust force, gas pressure and thermal load. Finite element analysis was used to analyze the cylinder liner model's performance. According to the numerical results, the maximum deflection for cast iron and steel was 0.135 and 0.109 mm, respectively, at the point of applied piston load. The maximum axial deflection for steel and cast iron was 0.67 mm, while the circumferential deflection was very tiny. Barve et al. [30] carried out finite element analysis on the various designs of the engine piston made up of various alloys of magnesium, graphene, aluminum and titanium. Structural and thermal analysis was performed in order to determine the thermal stress and damage due to applied pressure on the piston. Various stresses and strains were calculated along with the total deformation and heat fluxes. Concluded results showed that aluminum deforms substantially more than materials like graphene. Since graphene can withstand temperature changes inside an engine with ease, its higher thermal conductivity in heat flux tests may prove useful. Kumar et al. [31] examined the stresses developed on the cylinder liner of a diesel engine as a result of thermal load, thrust force, and pressure. In this work the thermal barrier coated or uncoated liner of a diesel engine using 0.15 mm thick NiCrAl as the bond coat and 0.35 mm thick CaZrO_3_ as the coating material was examined. The mechanical stress along the cylinder liner in terms of radial, longitudinal, and hoop stresses was calculated using finite element method. The variations in the von Misses stress, normal stress, stain and total deformation were watched for chosen ceramic materials. Results demonstrated that because of combined pressure and thermal loads, axial stress in cylinder liners was greater than radial and hoop stress. The maximum radial stress calculated at the free end of a conventional liner was 1.3706 × 10^6^ Pa.

The objective of this study is to optimize the heat transfer performance of three different motorcycle engine designs through a comprehensive fin heat transfer analysis. While the stated aim is to minimize external body temperature, the underlying rationale and practical significance of this goal are paramount.

Efficient heat dissipation plays a crucial role in maintaining the longevity and performance of motorcycle engines. Elevated temperatures can accelerate wear and tear, potentially leading to premature component failure and increased maintenance costs. Therefore, optimizing heat transfer can prolong engine lifespan and reduce maintenance requirements, thereby enhancing reliability and cost-effectiveness for motorcycle owners.

Minimizing external body temperature contributes to enhanced safety by mitigating the risk of heat-related injuries or discomfort to riders. Excessive heat can cause burns or discomfort, particularly in areas where riders come into contact with the motorcycle's surfaces. By optimizing heat transfer and reducing external temperatures, we aim to create a safer riding environment for motorcyclists and the engine's smooth operation throughout its lifespan. Minimizing an engine's external body temperature has real-world effects on performance, fuel economy, maintenance expenses, engine longevity, safety, emissions compliance, and resale value. This offers owners several advantages that make driving more affordable, dependable, and ecologically friendly.

The significance of analyzing fin heat transfer in motorcycle engine models extends to the scientific community in several ways. This work contributes to the understanding of heat transfer principles and their application in real-world engineering systems. By studying fin heat transfer in motorcycle engines, researchers expand scientific knowledge in thermal engineering, benefiting not only motorcycle design but also other fields reliant on heat management. The insights gained from this analysis inform best practices in engine design, particularly in optimizing cooling systems for improved performance and reliability. By identifying effective fin configurations, researchers provide practical guidance for engineers seeking to enhance heat transfer efficiency in various applications beyond motorcycles. Understanding the factors influencing fin heat transfer allows for innovation and optimization in engine design. Design of Experiments (DOE) methodology allows researchers to systematically explore the effects of different fin configurations and parameters on heat transfer performance. By conducting controlled experiments and analyzing the results, researchers can identify optimal design solutions that maximize cooling efficiency. The application of the Taguchi Method in fin heat transfer analysis enables robust design optimization by systematically evaluating the effects of design parameters on heat transfer performance while considering variations in operating conditions. By using orthogonal arrays and signal-to-noise ratios, the Taguchi Method facilitates efficient statistical analysis, allowing researchers to identify the most influential factors that help in achieving superior engineering outcomes within the scientific community.

## 2 Methodology

**Fig. 1:**
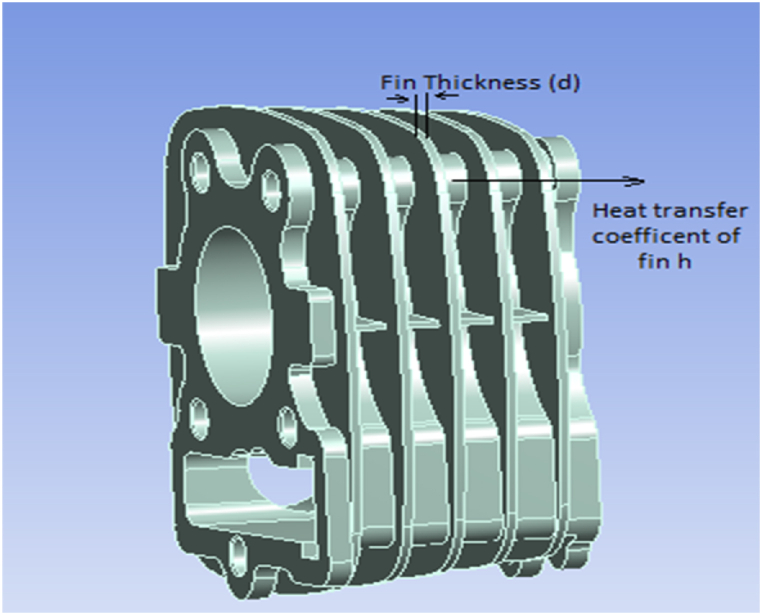
Schematic geometry of a radial fin array with convective heat transfer.

**Fig. 2:**
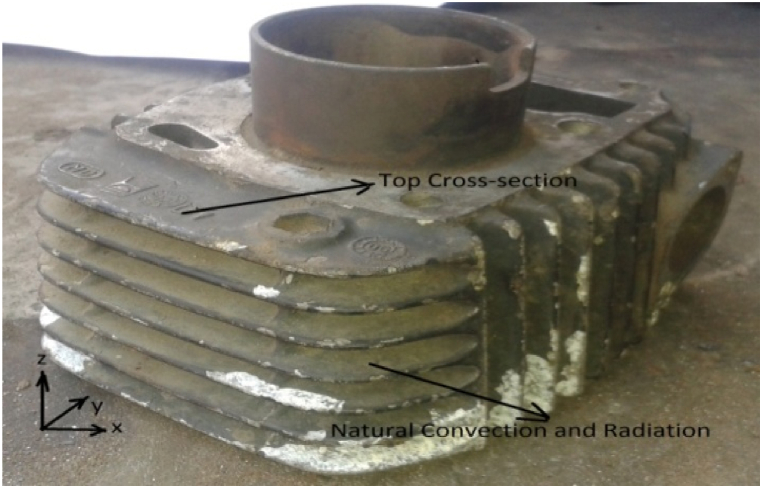
Fin Geometry with coordinate system.

Heat transfer over a finite temperature difference is typically what characterizes convective heat transfer events. These phenomena are regarded as irreversible thermodynamic processes. The schematic geometry of a radial fin array and fin geometry with a coordinate system are shown in Fig. 1, Fig. 2. The physics applied in the case of heat transfer by fins through forced convection is similar to the natural convection case. The primary step in the method of calculating the convection heat transfer coefficient, h is to determine the turbulent flow character conditions since the convection heat transfer coefficient depends strongly on which of these conditions exist.

The Reynolds number and Prandtl number varies with the wind velocity and can be expressed as:

(1) $$Re=\frac{\rho \times v\times D}{\mu }$$

(2) $$\mathrm{Pr}=\frac{c_{p}\times \mu }{k}$$

The Nusselt number depends on the value of the Prandtl number and can be expressed as:

(3) $$\begin{array}{cc} Nu=\frac{h\times D}{k}=0.664\times {Re}^{0.5}\times {\mathrm{Pr}}^{0.33} & \text{if}\;\mathrm{Pr}\;>=0.6 \end{array}$$

Substituting equation (1), (2) into Eq. (3), calculate the value of heat transfer coefficient.

In this study, no experimental work was carried out. The simulation was performed on three different companies' engines using ANSYS Workbench 14.5. The three different engine CAD geometry was created, meshing was performed, boundary conditions were applied, simulations were carried out and finally, the results were extracted.

In engines, the balance between convective heat transfer and air-cooled fin design is pivotal for efficient cooling and optimal performance. Fins integrated into components such as cylinder heads and engine blocks amplify the surface area, facilitating heat dissipation to surrounding air. Fin parameters like thickness, spacing, and shape are optimized to maximize heat transfer. Augmenting airflow over fins ensures effective convective heat transfer. By harmonizing these elements, engineers achieve an optimal balance, vital for reliable engine performance and longevity.

The three different designs of the engines of different companies were chosen as these companies are among the leading manufacturers of two-wheeler engines in India, each with its own unique engineering approaches, technologies, and market positioning. By selecting engines from these manufacturers, we can compare the design philosophies, performance characteristics, and technological innovations specific to each brand.

## 3 Description of the system

### 3.1 General Descriptions

Fig. 1 shows the system of the engine with a three-dimensional coordinate system. The engine is heated at the exhaust port because of exhaust passing through this port as shown in Fig. 2. The topmost part is the top cross-section area on which the cylinder head is mounted and the top cross-section cannot be opened in an environment. Fig. 2 shows the total area that participates in natural convection and radiation with a heat transfer coefficient related to wind velocity in the y direction.

### 3.2 Engine specification

The following Table 1 shows the specifications of three different engines.

**Table 1:** Engine specification [31].

S.No	Specification	Design A	Design B	Design C
1.	Type	Air-cooled 4 stroke single cylinder OHC	4 Stroke natural air-cooled	Air-cooled 4 stroke single cylinder
2.	Displacement	124.7 cc	134.21 cc	87.8 cc
3.	Max. Power	6.72 KW @ 7000 RPM	9.64 KW @ 8500 RPM	3.68 KW @ 6500 RPM
4.	Max. Torque	10.35 N-m @ 4000 RPM	8.6 Nm @ 5000 RPM	5.8 Nm @ 4000 RPM
5.	Bore * Stroke	52.4 * 57.8 mm	47 * 58.5 mm	51 * 73 mm

### 3.3 Material specifications

Most of the Engine manufacturing companies use aluminum alloy 6061 as a raw material, following are the properties shown in Table 2.

**Table 2:** Material specification [31].

Mechanical Properties	Al 6061
Thermal conductivity	300 (W/mmK)
Specific Heat	963 (J/kg ^o^C.)
Density	2.7 g/cc
Nodal Temperature	750 K
Bulk Temperature	283 K

## 4 Finite element analysis

**Fig. 3:**
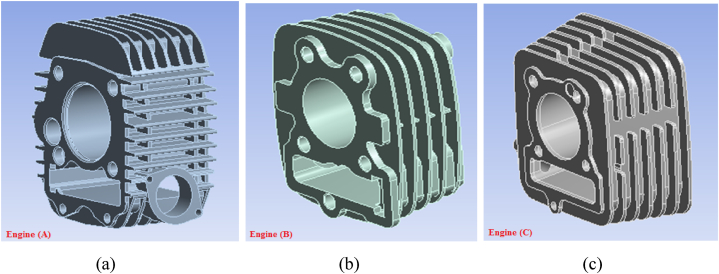
(a–c): Modeled different engines with fin.

Finite element analysis is a numerical approach for solving the many problems in engineering and daily life technology. The main advantage is that find out all parameters related to the product like as deformation, factor of safety, life, stress, etc. before the prototyping. In this monitoring performed a Finite element analysis on three different companies’ engines using ANSYS Workbench 14.5 and found out the maximum heat flux generated and temperature distribution in the fin. Fig. 3(a–c) shows the three different engine cylinder casing cad of the motorcycle with different fin radius, thickness, cross-sectional area, and end treatments.

**Fig. 4:**
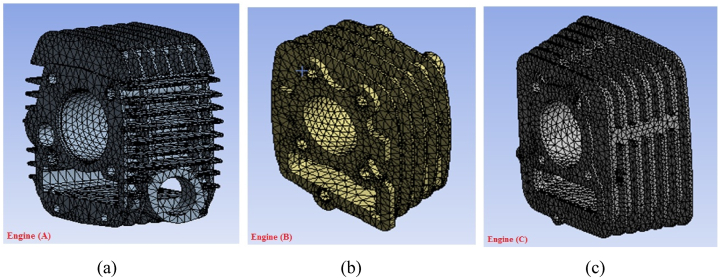
(a–c) Meshed fin geometry for triangular shape.

Fig. 4(a–c) shows the meshed structure of the developed model. The tetrahedron meshing was utilized for meshing the engine. During the meshing, the element sizes for engine A, B and C were taken 1.25 mm, 1.50 mm and 1.30 mm respectively which were determined by performing the mesh sensitivity analysis. The number of elements in engine A, B and C was 82342, 75448 and 79386 respectively.

### 4.1 Mesh sensitivity analysis

A mesh sensitivity analysis was performed for optimum mesh size selection and is shown in Table 3. The analysis was performed for the temperature of 750^o^C at the piston surface, velocity of 40 km/h and heat transfer coefficient of 50 W/m^2^K. In the mesh sensitivity analysis, the size of the mesh was varied from 3.50 mm to 1.00 mm. From Table 3 it was observed that after the element size of 1.25 mm for engine A, element size of 1.50 mm for engine B and element size of 1.30 mm for engine C there was not much variation in the outer temperature of engine A, B and C respectively. So, the 1.25 mm mesh for engine A, 1.50 mm mesh for engine B and 1.30 mm mesh for engine C were selected for further calculation.

**Table 3:** The mesh sensitivity analysis

Element Size (mm)	Outer Engine Temperature (^o^C)		
	Engine A	Engine B	Engine C
3.50	189.57	188.67	191.11
3.00	188.87	187.72	190.52
2.50	187.32	186.53	189.76
2.00	186.94	185.11	189.23
**1.50**	186.42	**184.31**	188.93
**1.30**	185.49	184.29	**187.22**
**1.25**	**185.39**	184.27	187.20
1.00	185.37	184.26	187.19

**Fig. 5:**
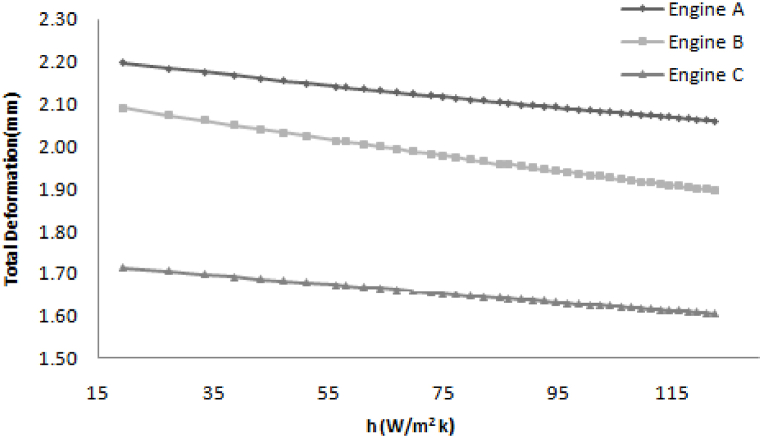
Variation in Total deformation with increasing heat transfer coefficient.

Fig. 5 shows that the heat transfer coefficient depends on the wind velocity. The lower deformation is a gain in the case of engine B so lower deformation is desired, so engine B is the optimum design.

**Fig. 6:**
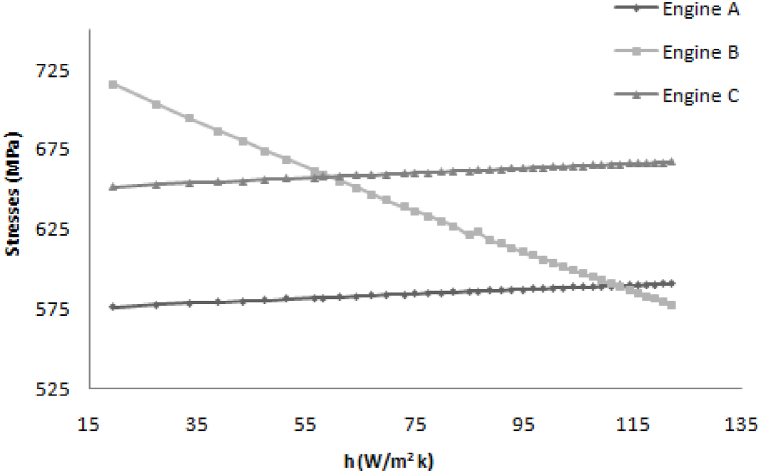
Variation in stresses with increasing heat transfer coefficient.

Fig. 6 shows that the heat transfer coefficient depends on the wind velocity. The lower normal stress is a gain in the case of engine B so normal stress is desired, so engine B is the optimum design.

**Fig. 7:**
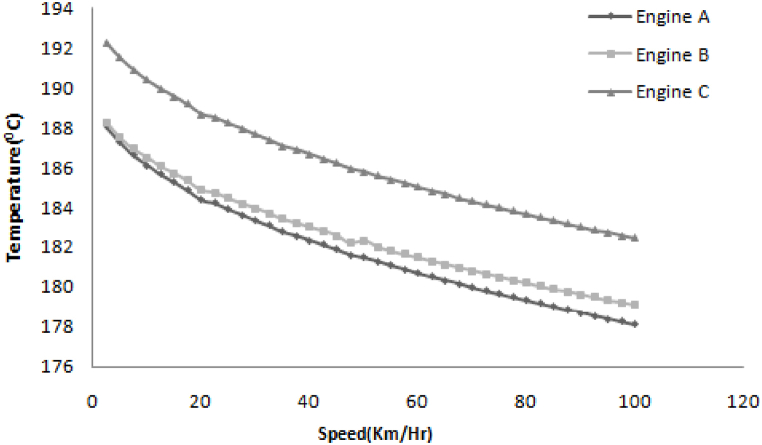
Variation in temperature with increasing speed.

Fig. 7 shows that the heat transfer coefficient depends on the wind velocity. The lower temperature is a gain in the case of engine B so a lower temperature is desired, so engine B is optimum design.

## 5 Design of Experiment

The Taguchi method was used in this study's experiment design. Robust parameter design makes use of Taguchi designs, where the main objective is to identify factor settings that minimize response variation while modifying (or maintaining) the process on target. A strong and effective technique for creating goods that function consistently and optimally under a range of circumstances is to use Taguchi designs. Designs are employed for robust parameter design, where the main objective is to identify factor configurations that minimize response variation while making necessary adjustments to the process to keep it on course. Taguchi offers a reliable and effective technique for creating goods that perform well and consistently under a range of circumstances. In this study, three parameters fin thickness, end treatment (chamfer, flat, fillet), and cross fins are taken. In the DOE analysis, the L9 orthogonal array was used in which three input factors of the engine were taken. From these three input factors, 9 cases were generated using MINITAB software. The simulations were performed on these 9 cases, and output i.e. temperature, deformation and stresses were generated.

The selection of parameters like fin thickness, size, and shape in motorcycle engine design is driven by their critical role in optimizing heat dissipation and thermal management. The selection of parameters was guided by their known influence on heat transfer performance. By systematically exploring these parameters through DOE, engineers can identify the most effective design configurations that balance cooling efficiency with other performance and reliability considerations. Table 4 shows the optimum case of the design of experiment.

**Table 4:** Design of experiment case

S.No	Thickness	End Treatment	Cross Fins
1	2	1	0
2	2	2	4
3	2	3	8
4	2.5	1	4
5	2.5	2	8
6	2.5	3	0
7	3	1	8
8	3	2	0
9	3	3	4

## 6 Results and discussion

### 6.1 Taguchi analysis

This concern shows the consequence of Taguchi analysis and surface response methods and optimization of the ranks of parameters that are used for design modification. This allows for the collection of the necessary data to determine which factors most affect product quality with a minimum amount of experimentation.

a. Minimum temperature versus Thickness, End treatment and Cross Fins

**Fig. 8:**
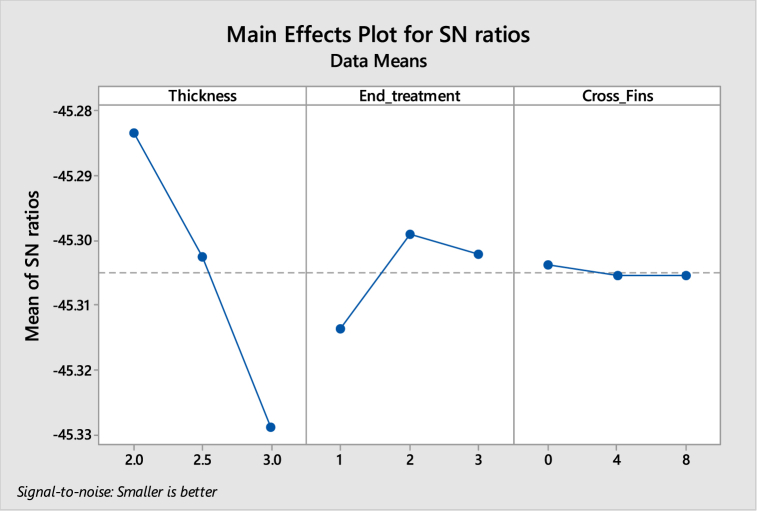
Response graph for signal to noise ratios.

**Fig. 9:**
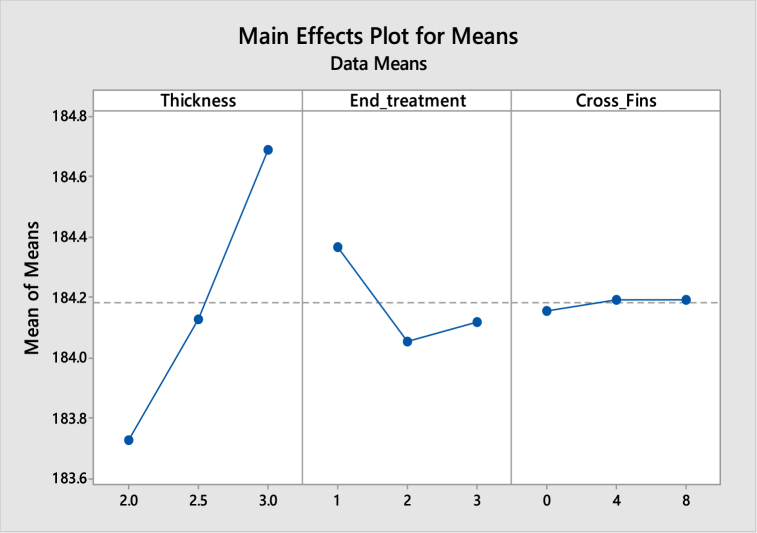
Response Graph for mean ratio.

Table 5, Table 6 shows the response table for Signal to Noise Ratios and mean ratio respectively. In the tables, the ranks of the input factors that affect the output i.e. temperature are given. Fig. 8, Fig. 9 show the response graphs for Signal to Noise ratios and mean ratio.

**Table 5:** Response table for signal to noise ratios

Level	Thickness	End treatment	Cross fins
1	−45.28	−45.31	−45.3
2	−45.3	−45.3	−45.31
3	−45.32	−45.3	−45.31
**Delta**	0.05	0.01	0
**Rank**	1	2	3

**Table 6:** Response Table for mean ratio

Level	Thickness	End treatment	Cross fins
1	183.7	184.4	184.2
2	184.1	184.1	184.2
3	184.7	184.1	184.2
**Delta**	1	0.3	0
**Rank**	1	2	3

Signal to noise ratio was a simple method to predict the effect of changing factors according to their levels to find the effect on product quality. In this study “smaller is better” was adopted as a quality indicator for the S/N ratio.

The best set of combination parameters should be determined by selecting the levels with high S/N ratio values from tables or graphs.

According to the temperature best set: **A3-B2-C3.**

b. Deformation versus Thickness, End treatment and Cross Fins

**Fig. 10:**
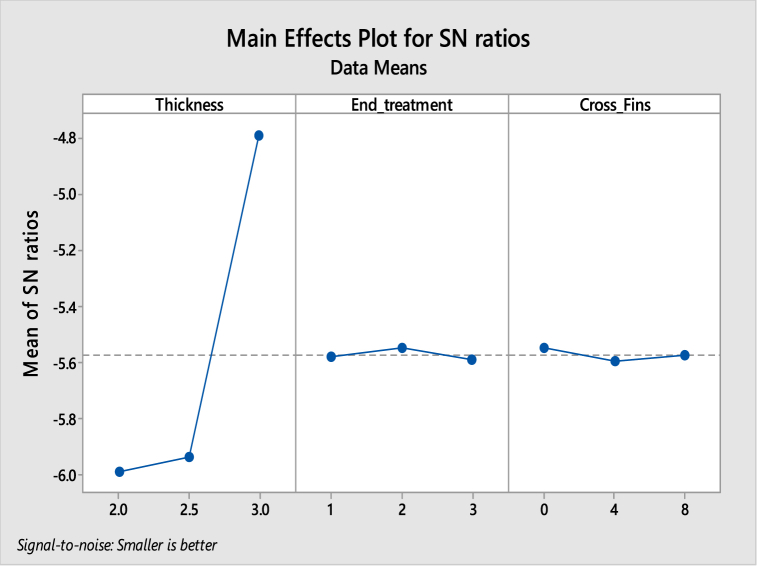
Response graph for signal to noise ratios.

**Fig. 11:**
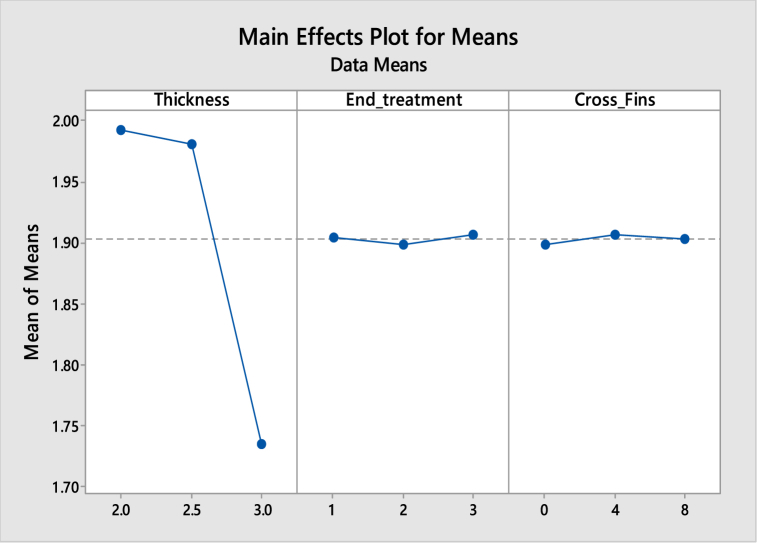
Response Graph for mean ratio.

Table 7, Table 8 shows the response table for Signal to Noise Ratios and mean ratio respectively. In the tables, the ranks of the input factors that affect the output i.e. deformation are given. Fig. 10, Fig. 11 show the response graphs for Signal to Noise ratios and mean ratio.

**Table 7:** Response table for signal to noise ratios

Level	Thickness	End treatment	Cross fins
1.	−5.992	−5.577	−5.549
2.	−5.938	−5.549	−5.595
3.	−4.787	−5.591	−5.573
Delta	1.205	0.042	0.046
Rank	1	3	2

**Table 8:** Response Table for mean ratio

Level	Thickness	End treatment	Cross fins
1.	1.993	1.905	1.899
2.	1.981	1.898	1.908
3.	1.735	1.907	1.904
Delta	0.258	0.008	0.009
Rank	1	3	2

Signal to noise ratio was the simple method to predict the effect of changing factors according to their levels to find the effect on product quality. In this study “smaller is better” was adopted as the quality indicator for the S/N ratio.

The best set of combination parameters should be determined by selecting the levels with high S/N ratio values from tables or graphs. According to the deformation best set: **A3-B2-C1.**

c. Normal stresses versus Thickness, End treatment and Cross Fins

**Fig. 12:**
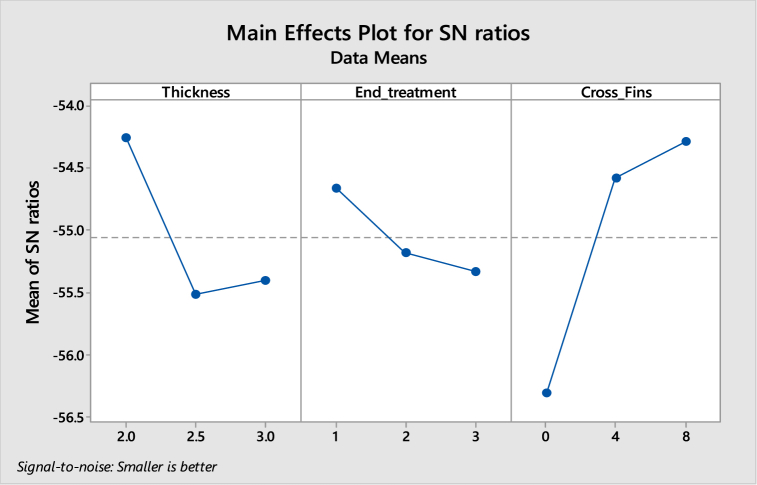
Response graph for signal to noise ratios.

**Fig. 13:**
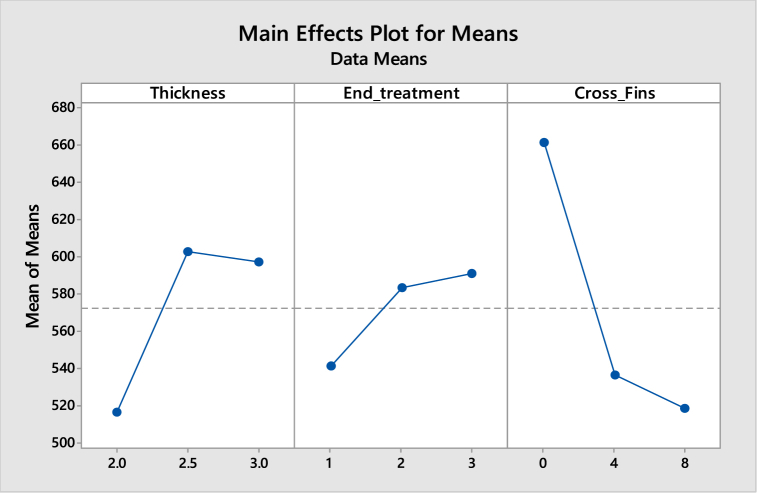
Response Graph for mean ratio.

Table 9, Table 10 show the response table for Signal to Noise Ratios and mean ratio respectively. In the tables, the ranks of the input factors that affect the output i.e. normal stresses are given. Fig. 12, Fig. 13 shows the response graphs for Signal to Noise ratios and mean ratio.

**Table 9:** Response table for signal to noise ratios

Level	Thickness	End treatment	Cross fins
1.	−54.25	−54.66	56.30
2.	−55.52	−55.17	54.58
3.	−55.40	−55.34	54.29
Delta	1.26	0.67	2.01
Rank	2	3	1

**Table 10:** Response Table for mean ratio

Level	Thickness	End treatment	Cross fins
1.	516.2	541.5	661.3
2.	603.0	583.6	536.7
3.	597.0	591.1	518.2
Delta	86.9	49.5	143.1
Rank	2	3	1

Signal to noise ratio was a simple method to predict the effect of changing factors according to their levels to find an effect on product quality. In this study “smaller is better” is adopted as a quality indicator for the S/N ratio.

The best set of combination parameters should be determined by selecting the levels with high S/N ratio values from tables or graphs.

According to normal stress best set: **A3-B3-C3.**

### 6.2 Surface response regression analysis

To investigate the connection between a response and a group of quantitative experimental variables or factors, response surface methods are utilized. When you have determined the critical few controllable factors and want to determine the factor settings that maximize the response, you frequently utilize these methods. Designs of this kind are typically selected when the response surface appears to be curved. The surface response parameter has been taken at a speed of 40 Km/h for engine B. The table shows the functional parameters by which the design is going to be optimized. Table 11 shows the values of the input and output factors.

a) Response Surface Regression: minimum temperature versus Thickness, End treatment and Cross Fins

**Table 11:** Surface response regression process parameters

S. No	Thickness	End Treatment	Cross Fins	Min. temp	Deformation	Normal stress
1.	2.0	2	4	183.49	1.99	501.19
2.	2.0	3	8	183.79	2.00	524.50
3.	2.5	1	4	184.44	1.99	580.32
4.	2.5	2	8	184.03	1.99	508.63
5.	2.5	3	0	183.92	1.97	720.08
6.	3.0	1	8	184.77	1.73	521.51
7.	3.0	2	0	184.65	1.72	740.98
8.	3.0	3	4	184.65	1.75	528.62
9	2.0	3	8	183.79	2.00	524.50

Table 12 shows the values of the adjusted sum of squares, adjust mean square, F-value, and P-value for the temperature for the linear model.

**Table 12:** Surface response regression for minimum temperature for the linear model

Source	DF	Seq SS	Contribution	Adj SS	Adj MS	F-Value	P-Value
**Model**	3	1.56372	90.55 %	1.56372	0.52124	15.96	0.005
**Linear**	3	1.56372	90.55 %	1.56372	0.52124	15.96	0.005
**Thickness**	1	1.50000	86.86 %	1.13626	1.13626	34.80	0.002
**End Treatment**	1	0.03273	1.90 %	0.01854	0.01854	0.57	0.485
**Cross Fins**	1	0.03100	1.79 %	0.03100	0.03100	0.95	0.375
**Error**	5	0.16328	9.45 %	0.16328	0.03266		
**Lack-of-Fit**	4	0.16328	9.45 %	0.16328	0.04082	*	*
**Pure Error**	1	0.00000	0.00 %	0.00000	0.00000		
**Total**	8	1.72700	100.00 %				

Table 13 shows that the S-value and R-value for the linear model have been valid only for h = 77.28 W/m^2^ k at 40 km/h.

**Table 13:** Model summary for minimum temperature

S	R-sq	R-sq(adj)	R-sq(pred)
0.180708	90.55 %	84.87 %	72.84 %

Regression Equation

Temp=181.680+1.012Thickness−0.0632EndTreatment+0.0204CrossFins

**Fig. 14:**
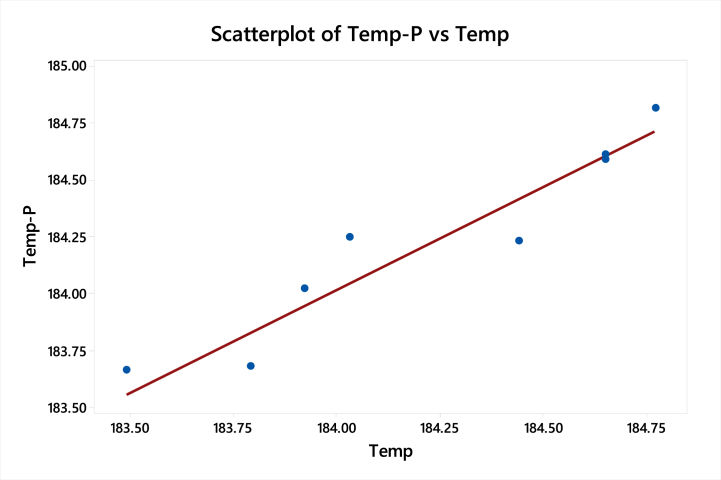
Curve fitting plot of temperature predicted and temperature for the linear model.

Fig. 14 shows the curve-fitting plot of the temperature predicted and temperature for the linear regression model. The graph shows that the points lie very close to the curve fitting line which shows that predicted results are accurate.

b) Response Surface Regression: Deformation versus Thickness, End treatment and cross-fins

Table 14 shows the values of the adjust sum of squares, adjust mean square, F-value, and P-value for the minimum deformation for the linear model.

**Table 14:** Surface response regression for minimum deformation for the linear model

Source	DF	Seq SS	Contribution	Adj SS	Adj MS	F-Value	P-Value
**Model**	3	0.105825	79.67 %	0.105825	0.035275	6.53	0.035
**Linear**	3	0.105825	79.67 %	0.105825	0.035275	6.53	0.035
**Thickness**	1	0.104017	78.31 %	0.089127	0.089127	16.51	0.010
**End Treatment**	1	0.001067	0.80 %	0.001444	0.001444	0.27	0.627
**Cross Fins**	1	0.000742	0.56 %	0.000742	0.000742	0.14	0.726
**Error**	5	0.026997	20.33 %	0.026997	0.005399		
**Lack-of-Fit**	4	0.026997	20.33 %	0.026997	0.006749	*	*
**Pure Error**	1	0.000000	0.00 %	0.000000	0.000000		
**Total**	8	0.132822	100.00 %				

Table 15 shows the S-value, and R-value for the linear model which have been valid only for h = 77.28 W/m^2^ K at 40 Km/h.

**Table 15:** Model summary for minimum deformation

S	R-sq	R-sq(adj)	R-sq(pred)
0.0734807	79.67 %	67.48 %	37.20 %

Regression Equation

Deformation=2.668−0.2835Thickness−0.0176EndTreatment−0.00316CrossFins

**Fig. 15:**
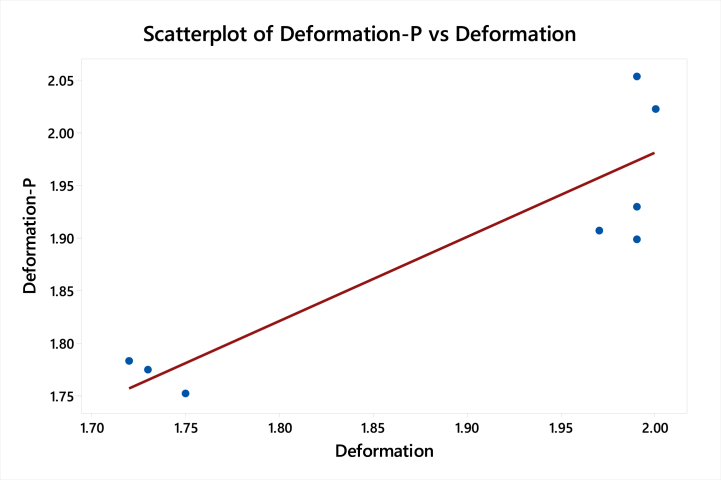
Curve fitting plot of deformation predicted and deformation for the linear model.

Fig. 15 shows the curve-fitting plot of deformation predicted and deformation for the linear regression model. The graph shows that the points do not lie very close to the curve fitting line which shows that predicted results are not accurate. Linear regression model is not showing better curve fitting so we need to migrate to nonlinear regression modeling.

Table 16 shows the values of the adjust sum of squares, adjust mean square, F-value, and P-value for the minimum deformation for the non-linear model.

**Table 16:** Surface response regression for minimum deformation for the non-linear model

Source	DF	Seq SS	Contribution	Adj MS	F-Value	P-Value
**Model**	6	0.132749	99.95 %	0.022125	608.44	0.002
**Linear**	3	0.105825	79.67 %	0.034839	958.06	0.001
**Thickness**	1	0.104017	78.31 %	0.061104	1680.35	0.001
**End Treatment**	1	0.001067	0.80 %	0.000161	4.44	0.170
**Cross Fins**	1	0.000742	0.56 %	0.000485	13.34	0.067
**Square**	3	0.026924	20.27 %	0.008975	246.81	0.004
Thickness*Thickness	1	0.026554	19.99 %	0.026250	721.88	0.001
End Treatment*End Treatment	1	0.000013	0.01 %	0.000000	0.01	0.931
Cross Fins*Cross Fins	1	0.000358	0.27 %	0.000358	9.85	0.088
**Error**	2	0.000073	0.05 %	0.000036		
**Lack-of-Fit**	1	0.000073	0.05 %	0.000073	*	*
**Pure Error**	1	0.000000	0.00 %	0.000000		
**Total**	8	0.132822	100.00 %			

Table 17 shows the S-value, and R-value for the Non-linear model which have been valid only for h = 77.28 W/m^2^ K at 40 Km/h.

**Table 17:** Model summary for minimum deformation

S	R-sq	R-sq(adj)	R-sq(pred)
0.0060302	99.95 %	99.78 %	–

Regression Equation

$$\text{Deformation}=\begin{array}{c} -0.532+2.2400\;\text{Thickness}+0.0050\;\text{End}\;\text{Treatment}+0.01023\;\text{Cross}\;\text{Fins}\\ -0.4982\;\text{Thickness}*\text{Thickness}+0.00045\;\text{End}\;\text{Treatment}*\text{End}\;\text{Treatment}\\ -0.000909\;\text{Cross}\;\text{Fins}*\text{Cross}\;\text{Fins} \end{array}$$

**Fig. 16:**
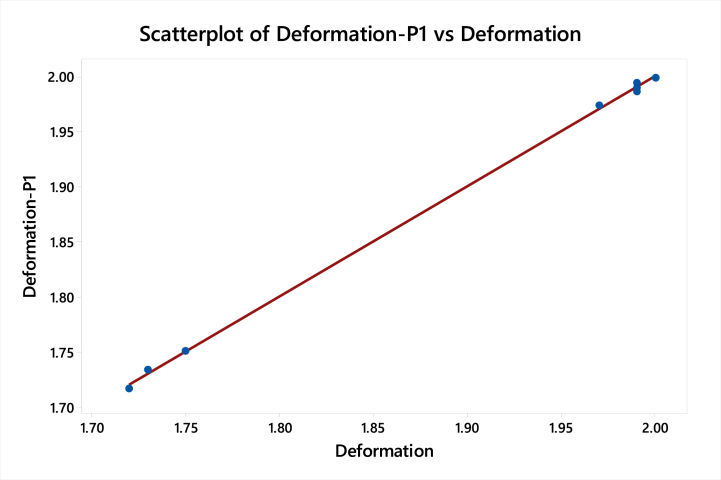
Curve fitting plot of deformation predicted and deformation for Non-linear model.

Fig. 16 shows the curve-fitting plot of deformation predicted and deformation for the non-linear regression model. The graph shows that the points lie very close to the curve fitting line which shows that predicted results are accurate.

c) Response Surface Regression: Normal stresses versus Thickness, End treatment and Cross Fins

Table 18 shows the values of the adjust sum of square, adjust mean square, F-value and P-value for the minimum normal stress for the non-linear model.

**Table 18:** Surface response regression for minimum normal stress for non-linear model

Source	DF	Seq SS	Contribution	Adj MS	F-Value	P-Value
**Model**	6	66594.3	97.18 %	11099.0	11.50	0.052
**Linear**	3	50582.6	73.82 %	18691.1	19.37	0.049
**Thickness**	1	9673.7	14.12 %	3.5	0.00	0.958
**End Treatment**	1	3545.6	5.17 %	632.9	0.66	0.503
**Cross Fins**	1	37363.3	54.53 %	41876.0	43.40	0.022
**Square**	3	16011.7	23.37 %	5337.2	5.53	0.157
Thickness*Thickness	1	765.7	1.12 %	42.8	0.04	0.853
End Treatment*End Treatment	1	158.4	0.23 %	981.8	1.02	0.419
Cross Fins*Cross Fins	1	15087.6	22.02 %	15087.6	15.64	0.058
**Error**	2	1929.7	2.82 %	964.8		
**Lack-of-Fit**	1	1929.7	2.82 %	1929.7	*	*
**Pure Error**	1	0.0	0.00 %	0.0		
**Total**	8	68523.9	100.00 %			

Table 19 shows that the S-value and R-value for the Non-linear model have been valid only for h = 77.28 W/m2 k at 40 km/h.

**Table 19:** Model Summery for minimum normal stress

S	R-sq	R-sq(adj)	R-sq(pred)
31.0617	97.18 %	88.74 %	*

Regression Equation

$$\text{Normal}\;\text{Stress}=\begin{array}{c} 731+99\;\text{Thickness}-110\;\text{End}\;\text{Treatment}-74.7\text{Cross}\;\text{Fins}\\ -20.1\;\text{Thickness}*\text{Thickness}+24.1\;\text{End}\;\text{Treatment}*\text{End}\;\text{Treatment}\\ +5.90\;\text{Cross}\;\text{Fins}*\text{Cross}\;\text{Fins} \end{array}$$

**Fig. 17:**
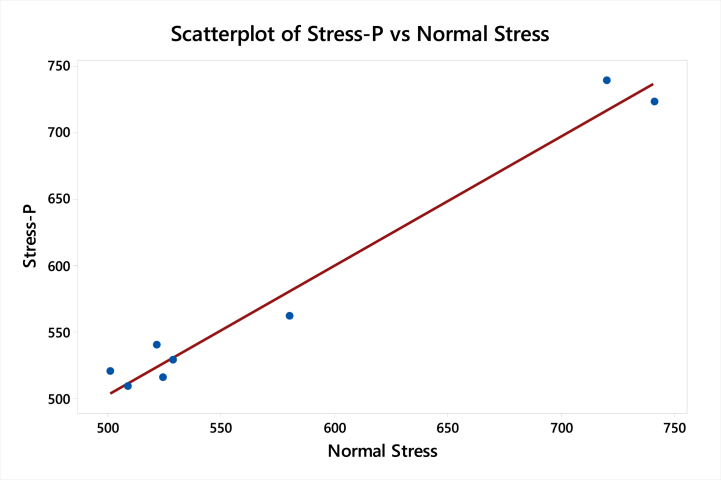
Curve fitting plot of Normal Stress predicted and Normal Stress for Non-linear model.

Fig. 17 shows the curve-fitting plot of Normal Stress predicted and Normal Stress for the non-linear regression model. The graph shows that the points lie very close to the curve fitting line which shows that predicted results are accurate.

The thermal conductivity of the material aluminium alloy 6061 is highly relevant to heat transfer in motorcycle engine design and has a significant impact on the results. Aluminium alloy 6061 is known for its relatively high thermal conductivity. It can efficiently transfer heat away from critical engine parts to the surrounding air. Higher thermal conductivity allows for faster heat dissipation, which is crucial for maintaining optimal operating temperatures in motorcycle engines. Aluminium alloy 6061 can enhance cooling performance and prevent overheating, thereby improving engine reliability and longevity.

The significance of the thickness, size, and shape of fins on the heat transfer performance of a motorcycle engine is profound and directly impacts the engine's efficiency, reliability, and longevity. Here's a breakdown of their significance:

**Thickness of Fin:** Optimizing fin thickness involves striking a balance between thermal conductivity and heat dissipation surface area. Thicker fins provide better cooling conditions.

**Size of Fin:** The size of fins directly affects the surface area available for heat transfer to the surrounding air. Larger fins typically offer greater heat dissipation capacity. By adjusting the size of fins, engineers can optimize the balance between heat dissipation efficiency and other design considerations.

**Shape of Fin:** The shape of the fins influences airflow patterns around the engine and affects heat transfer rates. Different fin shapes can alter the convective heat transfer coefficient. Optimizing fin shape involves the enhancement of convective heat transfer. Exploring various fin geometries achieves the cooling performance of the engine.

Overall, the significance of these fin parameters lies in their ability to regulate the engine's operating temperature, ensuring optimal performance and reliability. By carefully optimizing the thickness, size, and shape of fins, engineers can enhance heat transfer performance, minimize thermal stress on engine components, and prolong the engine's service life. Optimizing the thickness, size, and shape of fins in motorcycle engine design not only improves heat transfer performance but also has wide-ranging implications for engine efficiency, engine performance, engine lifespan, engine design innovation, reliability, rider comfort, and market competitiveness.

Analyzing the fin heat transfer of three distinct motorcycle engine models provides valuable insights into the thermal performance and design considerations of these engines. Here are some insightful remarks on such an analysis.

a) Conducting fin heat transfer analysis across multiple engine models allows for a comparative evaluation of their cooling efficiency. By quantifying parameters such as heat dissipation rates and temperature gradients, engineers can identify which engine design exhibits superior thermal performance under various operating conditions.
b) Each motorcycle engine model may employ different fin configurations to optimize heat transfer. By analyzing these variations, it becomes possible to identify the optimum design. The heat transfer analysis may reveal opportunities for optimizing fin design to enhance cooling performance. Identifying regions of high-temperature gradients or inefficient heat dissipation can inform targeted design modifications, such as adjusting fin thickness, size, or shape. Optimization efforts can lead to improved engine reliability, longevity, and performance.

Conducting fin heat transfer analysis on three distinct motorcycle engine models offers valuable insights into their thermal behavior and design optimization opportunities. These insights inform the iterative design process, leading to the development of more efficient, reliable, and performance-optimized motorcycle engines.

## 7 Conclusion and future scope

The findings of a fin heat transfer analysis of three distinct motorcycle engine models are presented in this study. Based on the investigation below mentioned observations are made.

1. The lower temperature gain was observed in the case of design B engine which concluded that engine B was of optimum design. Moreover, the lower deformation was gained in the case of engine B which indicated that engine B was of optimum design. Finally, the lower normal stress was gained in the case of engine B which showed that engine B was of optimum design.
2. The drawbacks of engine designs A and C were the higher temperature gain which hindered the engine's performance. Secondly, in engine designs A and C more deformation was observed in comparison to engine B. Finally, more normal stresses were developed in engine designs A and C in comparison to engine B.
3. The heat transfer rate was evaluated analytically and correlated to the minimum temperature at the cylinder head surface. The optimum geometry of a fin profile was obtained by maximizing the heat transfer rate. It was observed that the heat transfer coefficient depends on the wind velocity. As there was an increase in the wind velocity; the heat transfer coefficient increased. The heat transfer coefficient was dependent on the Reynolds number Nusselt number and Prandtl number.
4. The Taguchi method was implemented to watch the impact of three parameters fin thickness, end treatment (chamfer, flat, fillet), and the number of fins on minimum temperature, deformation, and normal stresses. Smaller is better was adopted as the quality indicator for the S/N ratio. Finally, the best-set results for the temperature, deformation, and normal stresses were calculated.
5. The value of R^2^ was calculated for the minimum temperature, deformation, and normal stresses. For minimum temperature, deformation and normal stresses the value of R^2^ comes out to be more than 70 % which shows a good indication of surface response regression analysis**.**
6. Finally, the regression equations were also developed for all process parameters in terms of thickness, end treatment, and the cross fins.

## Future scope

The future scope of studying motorcycle engine design and heat transfer analysis is promising, offering opportunities for further research and advancements in several areas. Some recommendations for future work are given below.

a) The effects of other parameters on the heat transfer of the engine can be studied in the future. The other parameters are piston design, cylinder head design, material selection, ambient temperature, etc.
b) Future research can expand to consider advanced optimization algorithms and techniques. This approach would enable us to explore a wider design space and identify optimal engine configurations that maximize performance, efficiency, and reliability.
c) The findings from this study can be extrapolated to other engine types and vehicles beyond motorcycles. Future research can apply similar heat transfer analysis techniques to other automotive engines and marine engines to improve overall efficiency and reliability.

## Data availability

**“**The datasets used and/or examined during the current study available from the corresponding author on reasonable request.”

## Funding

“This research was funded by 10.13039/501100023674Deanship of Research and Graduate Studies at King Khalid University, through the small group research under the Grant Number: (R.G.P.1/139/45).”

## CRediT authorship contribution statement

**Manish Dadhich:** Writing – original draft, Software, Methodology, Formal analysis, Conceptualization. **Vikas Sharma:** Writing – review & editing, Validation, Supervision, Investigation, Formal analysis. **Gaurav Jain:** Validation, Supervision, Resources, Investigation. **K. Loganathan:** Investigation, Formal analysis, Conceptualization. **V. Karunakaran:** Writing – review & editing, Software, Methodology, Data curation. **Mohamed Abbas:** Writing – review & editing, Supervision, Software, Project administration, Investigation, Funding acquisition, Data curation. **P. Subhashini:** Validation, Software, Resources, Methodology.

## Declaration of competing interest

The authors declare that they have no known competing financial interests or personal relationships that could have appeared to influence the work reported in this paper.
